# Mentorship needs at academic institutions in resource-limited settings: a survey at makerere university college of health sciences

**DOI:** 10.1186/1472-6920-11-53

**Published:** 2011-07-29

**Authors:** Damalie Nakanjako, Pauline Byakika-Kibwika, Kenneth Kintu, Jim Aizire, Fred Nakwagala, Simon Luzige, Charles Namisi, Harriet Mayanja-Kizza, Moses R Kamya

**Affiliations:** 1 Department of Medicine, Makerere University College of Health Sciences, P.O. Box 7072, Kampala, Uganda; 2 Uganda Fogarty Alumni Association, P.O. Box 7072, Kampala, Uganda; 3 Infectious Diseases Institute, Makerere University College of Health Sciences, P.O. Box 22418, Kampala, Uganda

**Keywords:** Mentorship, capacity building, health care delivery, research, academic institutions, Africa

## Abstract

**Background:**

Mentoring is a core component of medical education and career success. There is increasing global emphasis on mentorship of young scientists in order to train and develop the next leaders in global health. However, mentoring efforts are challenged by the high clinical, research and administrative demands. We evaluated the status and nature of mentoring practices at Makerere University College of Health Sciences (MAKCHS).

**Methods:**

Pre-tested, self-administered questionnaires were sent by email to all Fogarty alumni at the MAKCHS (mentors) and each of them was requested to complete and email back the questionnaire. In addition to training level and number of mentors, the questionnaires had open-ended questions covering themes such as; status of mentorship, challenges faced by mentors and strategies to improve and sustain mentorship within MAKCHS. Similarly, open-ended questionnaires were sent and received by email from all graduate students (mentees) registered with the Uganda Society for Health Scientists (USHS). Qualitative data from mentors and mentees was analyzed manually according to the pre-determined themes.

**Results:**

Twenty- two out of 100 mentors responded (14 email and 8 hard copy responses). Up to 77% (17/22) of mentors had Master's-level training and only 18% (4/22) had doctorate-level training. About 40% of the mentors had ≥ two mentees while 27% had none. Qualitative results showed that mentors needed support in terms of training in mentoring skills and logistical/financial support to carry out successful mentorship. Junior scientists and students reported that mentorship is not yet institutionalized and it is currently occurring in an adhoc manner. There was lack of awareness of roles of mentors and mentees. The mentors mentioned the limited number of practicing mentors at the college and thus the need for training courses and guidelines for faculty members in regard to mentorship at academic institutions.

**Conclusions:**

Both mentors and mentees were willing to improve mentorship practices at MAKCHS. There is need for institutional commitment to uphold and sustain the mentorship best practices. We recommend a collaborative approach by the stakeholders in global health promotion to build local capacity in mentoring African health professionals.

## Background

Mentoring, defined as a partnership in personal and professional growth and development, is a core component of medical education and career success [1,2]. However, mentoring efforts are challenged by high clinical, research and administrative demands on the mentors [3,4]. These challenges are underscored in developing countries where faculty members are overwhelmed with clinical roles as well as teaching and administrative responsibilities. Furthermore, mentoring practices are yet to take root at many academic institutions in resource-limited settings.

Currently, there is global emphasis to increase mentorship of young scientists in order to train the next leaders in global health [2,5]. In Uganda, the need for effective mentors has gradually risen over the past 5 to 10 years with the increasing number of well-trained professionals entering the work-force. Makerere University College of Health Sciences (MAKCHS), the largest medical school in the country, is taking lead in establishing institutionalized mentorship together with other local and international stakeholders in promoting excellence in education, research and health care delivery [6]. Once established, there is need to ensure sustainability of mentoring best practices at African academic institutions. It is in this context that this study was designed as a needs assessment survey to identify the gaps in mentoring practices at MAKCHS that can be addressed in an effort to create an academy of mentors in the region.

Using quantitative and qualitative methods, we set out to; 1) determine the status of mentoring practices, 2) describe the perceptions of mentors and mentees and 3) identify the challenges and recommendations of mentors and mentees in regard to effective mentoring at MAKCHS. Results from this study provide baseline data on mentoring needs that are critical to the formulation and sustainability of academic mentoring programs at academic institutions in sub-Saharan Africa (SSA).

## Methods

### Study population

This study was conducted by the Uganda Society for Health Scientists (USHS) and the Uganda Fogarty Alumni Association (UFAA). USHS consists of at least 700 Ugandan scientists in the fields of but not limited to basic sciences, health systems, clinical care and behavioral sciences. USHS has contacts for all current graduate students that have attended at least one USHS-organized meeting. UFAA, an interest group within USHS, consists of up to 180 Fogarty alumni that are part of USHS of whom majority (60%) are faculty members at MAKCHS. Both teams have junior and senior scientists and provide several opportunities for mentoring, dissemination of research findings, networking, and training in research related fields. The study population was a convenience sample of the Fogarty alumni that are faculty members at MAKCHS.

### Study procedures

This was a qualitative study using self-administered open-ended questionnaires that were completed by a convenience sample of MAKCHS faculty members (mentors) and graduate students (mentees) registered with USHS. Pre-tested, self-administered questionnaires for mentors were sent by email to all Fogarty alumni at the MAKCHS (mentors) and each of them was requested to complete and email back the questionnaire. Follow up phone calls were made to faculty members that did not respond to emails to whom hard copies were hand-delivered and collected after completion. In addition to demographic data, training and number of mentors, the questionnaires had open-ended questions covering themes such as; status of mentorship, challenges faced by mentors and strategies to improve and sustain mentorship within MAKCHS. Similarly, open-ended questionnaires were sent to and received by email from all graduate students (mentees) registered with USHS. Themes covered in the mentee's questionnaire included status of current mentor-mentee relationships, challenges faced and possible solutions to improve mentor mentee relationships. Completion of each questionnaire required at least 15 minutes. Qualitative data from mentors and mentees was analyzed manually according to the themes pre-determined by expert opinion of the seven-member-UFAA steering committee.

## Results

### Mentors characteristics and needs at MAKCHS

Twenty- two out of 100 mentors responded (14 email and 8 hard copy responses). Majority of the mentors were within the ages of 30-49 years and 68% were of male gender. Up to 77% (17/22) of the available mentors had Master's level training and only 18% (4/22) had doctorate level training. The current mentors are involved in research in the areas of clinical medicine, basic sciences, health systems as well as behavioral sciences and their areas of mentorship included clinical medicine, medical ethics, professionalism and research. About 40% of the mentors had two or more mentees while 27% had none. It was noted that mentors needed support in terms of training in mentorship skills and logistical/financial support to carry out successful mentorship (see Table 1).

**Table 1:** Status of mentorship practices at Makerere University College of Health Sciences

	Mentors	N = 22 respondents n (%)
**Age group (years)**	30-39	10 (46)
	40-49	8 (36)
	50-60	4 (18)
		
**Gender**	Male	15 (68)
		
**Level of education**	Masters degree	17 (77)
	Doctorate	4 (18)
	Bachelors	1 (5)
		
Principal area of research*	Clinical Medicine	10 (44)
	Health Sciences	7 (30)
	Basic sciences	16 (31)
		
Areas of mentorship*		
	Professionalism	13 (33)
	Clinical Medicine	10 (25)
	Research field	17 (43)
		
Number of mentees at the moment*		
	One	7 (37)
	Two and more	9 (47)
	None	3 (16)
		
**Number of mentees** one can handle in a year*		
	Less than five	18 (82)
	Five to Ten	3 (14)
	More than Five	1 (5)
		
Kind of support needed*	Training	20 (41)
	Logistical support	15 (31)
	Financial support	14 (29)

*Some mentors gave multiple responses

### Perspectives of mentees

We had 9 mentee respondents (5 emails and 4 hard copies). All mentees were within the 20-30-year age group and 8 out of 9 were of male gender. In general, mentees reported that mentorship is not yet institutionalized and it is currently occurring in an adhoc manner. This was presented by some of the respondents as lack of interest in career development. They said, *"Mentorship is not taken seriously by mentors and mentees"*, *"We do not have as much opportunity to be mentored until we get jobs to work in the research setting", Mentorship in the school of medicine is mentee-driven", "Mentorship is currently not well organized", "Mentorship starts very late when the program is about to end"*. The mentees mentioned challenges of limited time that is devoted to mentorship and limited time for mentor-mentee meetings. *"Our mentors are usually very busy and not available'*, *"There is a severe lack of time to meet my mentor", they reported*. Mentees expressed the lack of awareness of their roles as mentees not to mention the roles of their mentors. *"I meet my mentor when I have a problem, and it is not clear what my roles as a mentee are and what my mentor's roles are", "I have supervisors but I really don't consider them mentors because they are only concerned about my academic performance", they reported*. In regard to the proposed solutions, the mentees recommended that the institution should endeavor to facilitate mentor-mentee matching. They suggested, *"There is need to provide opportunities for interaction between mentors and mentees to support initiation of mentor-mentee relationships", "Students/junior scientists should be encouraged to actively approach mentors of their choice"*. In addition, mentees recommended improvement of mentoring skills among mentors and supervisors at the college (see Table 2). They reported, *"Mentees should be trained on their roles and expectations in the mentor-mentee relationships", "Mentors focus mainly on the research and forget other aspects of our lives", "Mentorship should include aspects of management, personal life, finances, etc"*.

**Table 2:** Mentees' perspectives about the status of mentoring practices at Makerere University College of Health Sciences.

Theme	Responses
**Status of mentorship**	Lack of specific academic mentorship program
	
**Challenges facing mentorship practices**	Adhoc method of choosing mentors
	Limited time allocated to mentorship
	Limited awareness of roles of mentors/mentees
	
**How mentorship can be improved**	Facilitation of mentor-mentee matching
	Improvement of mentoring skills

### Perspectives of the mentors

Overall, the mentors mentioned that there were very few available and/or practicing mentors at the college. One mentor mentioned, *"We have very few available mentors"*. Another mentor mentioned, *"Most of the post graduate students approach me personally for help with writing proposals, theses, abstracts etc; and often the demand becomes too overwhelming for me to handle"*. The mentors also mentioned that there was need for training courses and guidelines for faculty members/potential mentors in regard to mentorship in academic institutions. *"Mentors need guidelines and courses on how to mentor"*, "Mentors do not know what is expected of them", *"Mentors and mentees should know what is expected of them", they reported*. Similarly, they noted the lack of a mentorship program at the college together with the limited personnel, skills and logistics to carry out effective mentorship. *"There is no system within the school of Medicine to sustain the program", "We need to be facilitated to have our mentor-mentee activities in order to have better results", two mentors reported*.

The mentors recommended establishment of an academic mentorship program within the institution as reported, *"Institutionalize mentorship and put systems in place to sustain it" *and they encouraged strategies to initiate mentor-mentee relationships.

"*There is need to encourage initiation of mentor-mentee relationships", "Provide opportunities for professional interaction to share skills in mentorship, learn from others and improve mentoring skills", "The college teachers should encourage the juniors to pick interest in career development"*. Similar to mentees, mentors recommended improvement of mentoring skills; "*Equip mentors with mentorship skills", "Train mentors on the process of mentoring and mentoring best practices*". In regard to motivating mentors, they mentioned the need for institutional recognition of faculty members that have mentored many people. "The institution should recognize people that have mentored many people". The mentors proposed resource mobilization as a strategy to sustain mentorship; *"Mentors should be trained to compete for grants that can help to sustain mentorship activities" *(see Table 3).

**Table 3:** Perspectives of mentors (faculty members) at Makerere University College of Health Sciences about the status of mentorship

Theme	Responses
**Status of mentorship**	Low critical mass of mentors
	Limited mentorship skills
	
**Challenges facing mentorship practices**	Academic mentorship program at the college
	Initiation of mentor-mentee relationships
	Limited awareness of roles of mentors/mentees
	Lack of logistics for mentorship program
	Limited interest by mentees in pursuing academic careers
	
**How mentorship can be improved**	Establishment of an academic mentorship program at the college
	Improvement of mentoring skills
	Motivation of mentors by institutional recognition
	Resource mobilization to support mentoring activities

## Discussion

We found that majority of the faculty members at MAKCHS had adequate training and experience in basic sciences, clinical medicine, epidemiology and related fields; all of which make them suitable to mentor undergraduate and graduate students as well as colleagues. In general, both mentors and mentees mentioned few numbers of mentors, limited mentoring skills, lack of time to meet and lack of logistical support as the main challenges. The recommendations made on how to improve mentorship included; institutional support to set up an academic mentoring program at the College, facilitation of mentor-mentee matching processes, training mentees and mentors on their roles and responsibilities and motivation of mentors by institutional recognition.

### Limited pool of mentors

Up to 40% of mentors had ≥ two mentees while 27% had none. Results from this survey are comparable to reports from a systematic review where up to 20% of faculty members did not have a mentor [1]. Our data reflects a limited pool of mentors and this is supported by the qualitative data where some mentors feel overwhelmed by a big number of mentees. It is therefore critical for the College to explore all possible strategies to increase the critical mass of mentors. One possible strategy is to redefine the roles and responsibilities of faculty members and re-emphasize the fact that faculty members/scientists/teachers/clinicians have professional obligation to educate and mentor the next generation of the profession's practitioners [2]. The USHS and UFAA have a consortium of scientists that could be supported to develop mentoring skills and contribute to the pool of mentors at MAKCHS. However, there is need to address other challenges that include retaining physicians in academic medicine and dedication of adequate resources to support teaching and scholarly work.

### Improvement of mentorship skills

Both mentors and mentees expressed the need to acquire the actual skills to establish and sustain fruitful mentor-mentee relationships. This is probably why mentees were dissatisfied with the time allocated to them by mentors while the mentors were also dissatisfied with the low enthusiasm shown by mentees. Similarly, some mentees were concerned that their mentorship was limited to academic performance with limited or no attention to individual well being. This implies the need for attitude change with an effort to make mentor-mentee relationships mutually beneficial. There is evidence, from other academic mentoring programs, to show that the mentor's roles range from informal personal support to formalized mentorship relationships [1,7,8]. Thus the need to diversify the scope of mentoring activities to include medical ethics, professionalism, personal support, research implementation, dissemination and translation into policy in order to impact health policies and service delivery and maximize the benefits to the mentors, mentees and the entire community. The senior leadership and faculty members should get involved in the schools' mentorship program to train, explore and promote effective mentoring skills [9].

We encourage MAKCHS and other African academic institutions to invest in innovative ways to implement and expand the utilization of the recently launched 'Mentor's manual for health sciences training in Africa' [6]. The Academic Alliance for AIDS care and prevention in Africa and the Botswana-Harvard AIDS Institute Partnership for HIV Research and Education are examples of models where local and international collaborations have partnered to train and mentor African scientists in HIV/AIDS care through clinical care and research scholarships [5,10]. These provide innovations and strategies that can be utilized to enrich mentorship practices at academic institutions in similar settings. There is need to apply and expand these capacity building models beyond HIV/ADS care to improve mentorship practices in all relevant fields at African academic institutions.

### Protected time for mentorship

There was a general outcry by both mentors and mentees that there was limited time to devote to mentorship activities. This is probably due to 1) the limited number of available mentors for the large number of young scientists that need mentors and 2) the lack of protected time allocated to mentorship since the senior scientists are busy struggling to balance responsibilities of teaching, clinical care and research. There is evidence that mentorship programs are improved through provision of protected time to faculty members to participate in career development efforts such as the mentoring program [2,11]. In addition, faculty members should be encouraged to participate actively in scholarly activities to develop academic skills and credibility for competitive international grants [5] to support capacity building and mentorship. Thus, the need for regular training of faculty members in scientific writing and competitive 'grantsmanship' in order to maximize the benefits of both mentors and mentees in regard to career development [11].

### Logistical support for mentorship

It was mentioned that mentors needed logistical support to effectively perform their mentorship activities. There is need for academic institutions to identify and propose strategies to overcome the economic and organizational barriers to effective mentoring by all faculty members [11]. This study did not provide details on how current mentoring activities are conducted, monitored and evaluated in the absence of a formal mentoring program at MAKCHS. Effective mentorship is pertinent to excellence in medical education, research and service delivery; therefore, operational studies are required in this area. More so to quantify the mentoring needs in terms of personnel, time and cost requirements for an effective mentoring program [1] within the constraints of limited resources in sub-Saharan Africa.

The main limitation for this study was the low response rate of the scientists that were contacted to participate in the survey despite attempts to follow them up with reminders. Follow-up telephone reminders were made to participants that did not respond to emails and these were followed with printed questionnaires and reminders prior to collection of the completed questionnaires. The low response could reflect the low priority that mentorship activities take amidst the overriding patient care, teaching and administrative needs thereby amplifying the need to re-emphasize mentorship despite the busy schedules of faculty members.

## Conclusion

Both mentors and mentees are willing to improve mentorship practices at MAKCHS. There is need for institutional commitment to uphold and sustain the mentoring best practices for the next generation of leaders in health and related fields. We recommend a collaborative approach by local and international stakeholders in global health promotion to design and test innovative ways of building local capacity in mentoring African health professionals in order to develop leaders that will mitigate the challenges facing global health.

## Competing interests

The authors are beneficiaries of the FIC that provided the funding to conduct this study. However, FIC did not contribute to the design, analysis, interpretation of data and the decision to publish this paper.

## Authors' contributions

DN, PB, FN, CN, JA, SL and KK participated in the conception of the study, analysis and interpretation of the results. DN drafted the manuscript. HM and MK provided leadership to the conception of the study and interpretation of results. All authors reviewed the manuscript and gave approval of the final version to be published.

## Pre-publication history

The pre-publication history for this paper can be accessed here:

http://www.biomedcentral.com/1472-6920/11/53/prepub
